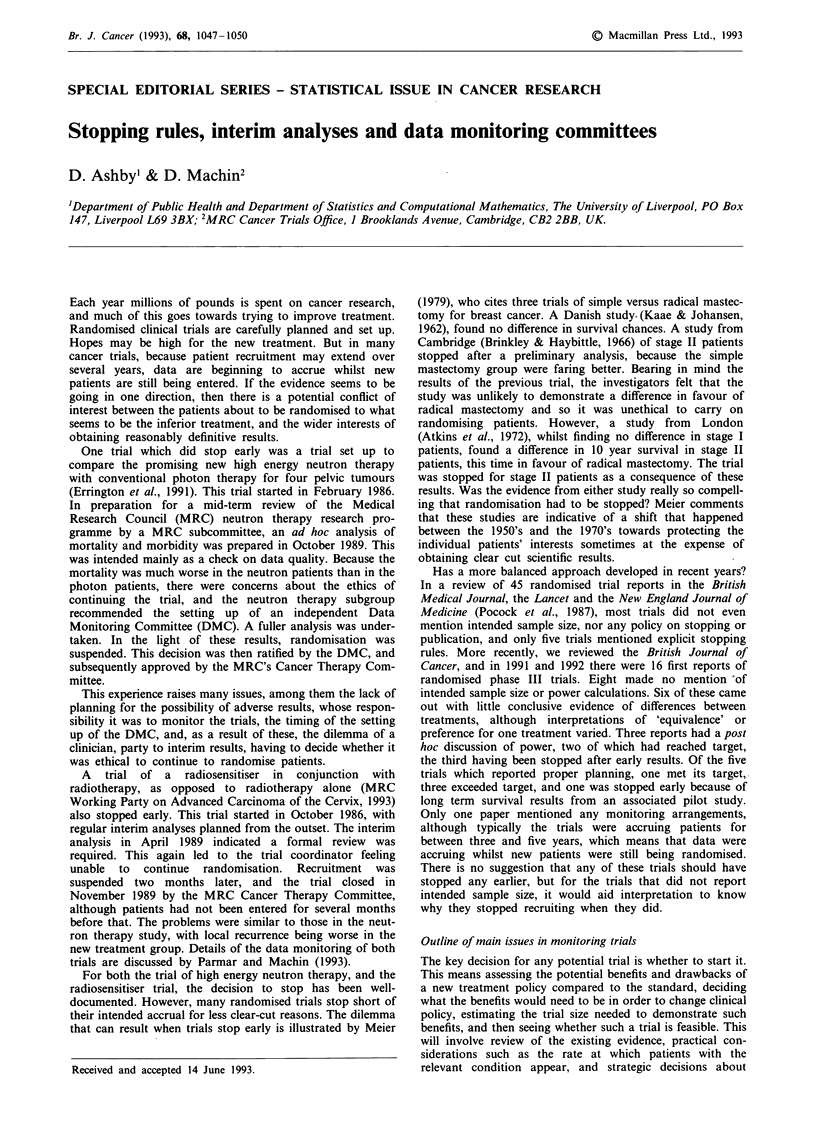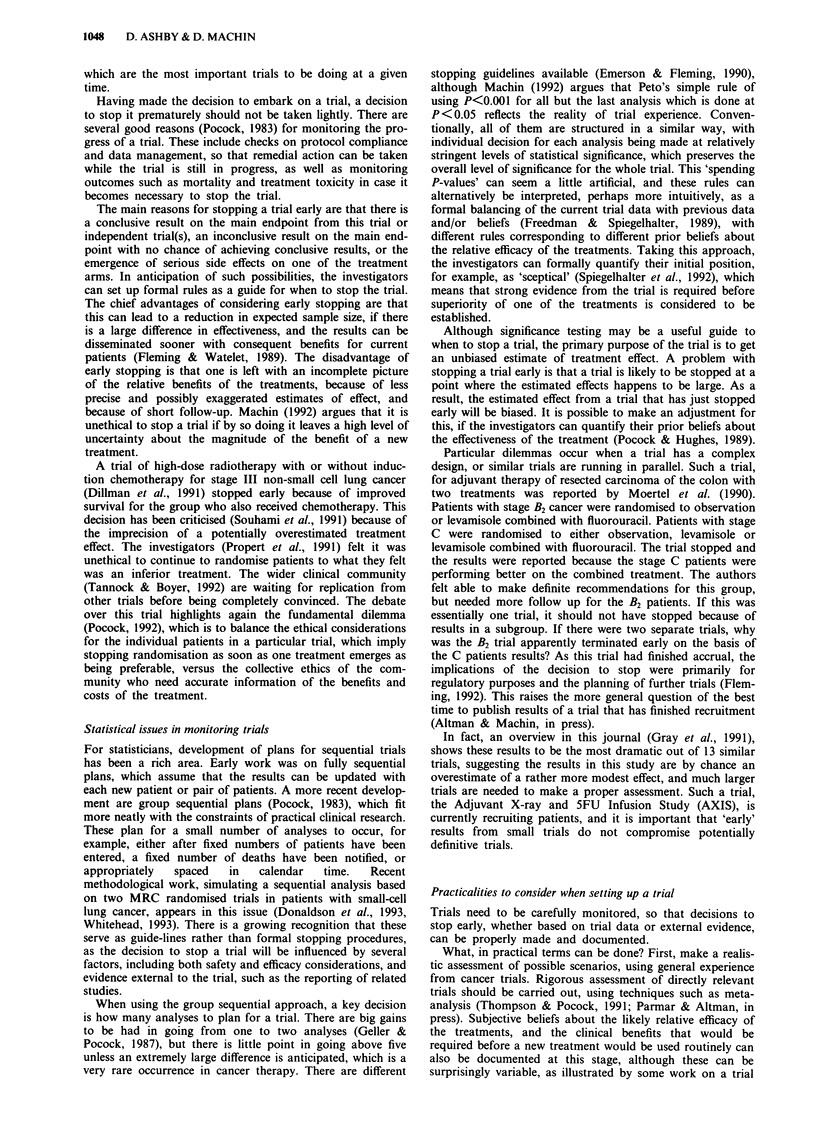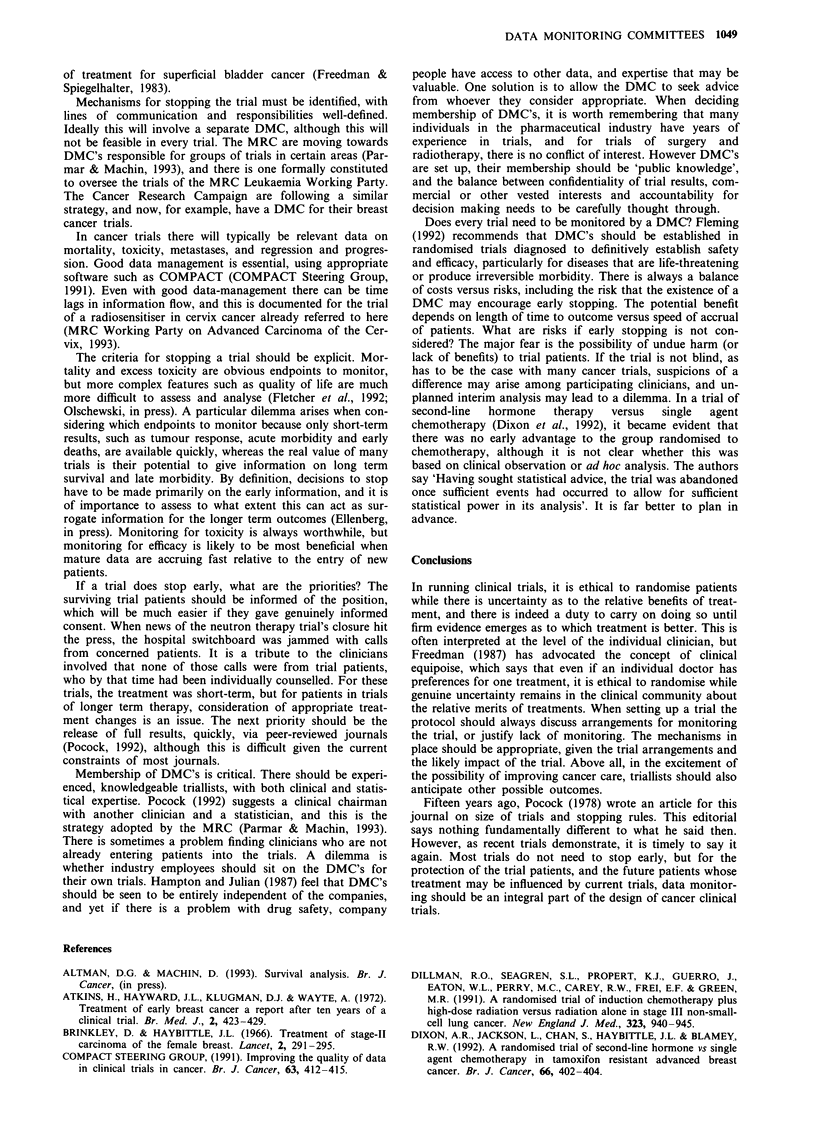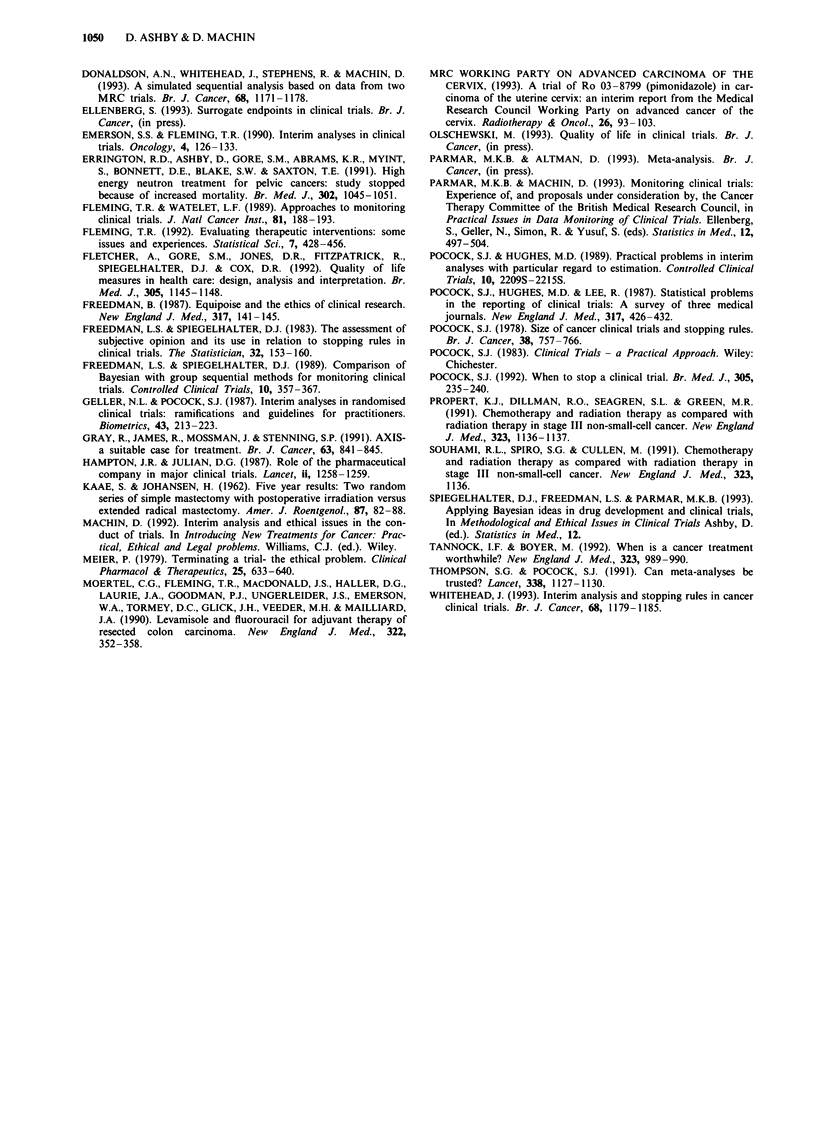# Stopping rules, interim analyses and data monitoring committees.

**DOI:** 10.1038/bjc.1993.481

**Published:** 1993-12

**Authors:** D. Ashby, D. Machin


Br. J. Cancer (1993), 68, 1047–1050 © Macmillan Press Ltd., 1993

SPECIAL EDITORIAL SERIES – STATISTICAL ISSUE IN CANCER RESEARCH

### Stopping rules, interim analyses and data monitoring committees

D. Ashby[1] & D. Machin[2]

[1]*Department of Public Health and Department of Statistics and Computational Mathematics, The University of Liverpool, PO Box 147, Liverpool L69 3BX;* [2]*MRC Cancer Trials Office, 1 Brooklands Avenue, Cambridge, CB2 2BB, UK.*

Each year millions of pounds is spent on cancer research, and much of this goes towards trying to improve treatment. Randomised clinical trials are carefully planned and set up. Hopes may be high for the new treatment. But in many cancer trials, because patient recruitment may extend over several years, data are beginning to accrue whilst new patients are still being entered. If the evidence seems to be going in one direction, then there is a potential conflict of interest between the patients about to be randomised to what seems to be the inferior treatment, and the wider interests of obtaining reasonably definitive results.

One trial which did stop early was a trial set up to compare the promising new high energy neutron therapy with conventional photon therapy for four pelvic tumours (Errington *et al.*, 1991). This trial started in February 1986. In preparation for a mid-term review of the Medical Research Council (MRC) neutron therapy research programme by a MRC subcommittee, an *ad hoc* analysis of mortality and morbidity was prepared in October 1989. This was intended mainly as a check on data quality. Because the mortality was much worse in the neutron patients than in the photon patients, there were concerns about the ethics of continuing the trial, and the neutron therapy subgroup recommended the setting up of an independent Data Monitoring Committee (DMC). A fuller analysis was undertaken. In the light of these results, randomisation was suspended. This decision was then ratified by the DMC, and subsequently approved by the MRC's Cancer Therapy Committee.

This experience raises many issues, among them the lack of planning for the possibility of adverse results, whose responsibility it was to monitor the trials, the timing of the setting up of the DMC, and, as a result of these, the dilemma of a clinician, party to interim results, having to decide whether it was ethical to continue to randomise patients.

A trial of a radiosensitiser in conjunction with radiotherapy, as opposed to radiotherapy alone (MRC Working Party on Advanced Carcinoma of the Cervix, 1993) also stopped early. This trial started in October 1986, with regular interim analyses planned from the outset. The interim analysis in April 1989 indicated a formal review was required. This again led to the trial coordinator feeling unable to continue randomisation. Recruitment was suspended two months later, and the trial closed in November 1989 by the MRC Cancer Therapy Committee, although patients had not been entered for several months before that. The problems were similar to those in the neutron therapy study, with local recurrence being worse in the new treatment group. Details of the data monitoring of both trials are discussed by Parmar and Machin (1993).

For both the trial of high energy neutron therapy, and the radiosensitiser trial, the decision to stop has been well-documented. However, many randomised trials stop short of their intended accrual for less clear-cut reasons. The dilemma that can result when trials stop early is illustrated by Meier (1979), who cites three trials of simple versus radical mastectomy for breast cancer. A Danish study (Kaae & Johansen, 1962), found no difference in survival chances. A study from Cambridge (Brinkley & Haybittle, 1966) of stage II patients stopped after a preliminary analysis, because the simple mastectomy group were faring better. Bearing in mind the results of the previous trial, the investigators felt that the study was unlikely to demonstrate a difference in favour of radical mastectomy and so it was unethical to carry on randomising patients. However, a study from London (Atkins *et al.*, 1972), whilst finding no difference in stage I patients, found a difference in 10 year survival in stage II patients, this time in favour of radical mastectomy. The trial was stopped for stage II patients as a consequence of these results. Was the evidence from either study really so compelling that randomisation had to be stopped? Meier comments that these studies are indicative of a shift that happened between the 1950's and the 1970's towards protecting the individual patients' interests sometimes at the expense of obtaining clear cut scientific results.

Has a more balanced approach developed in recent years? In a review of 45 randomised trial reports in the *British Medical Journal*, the *Lancet* and the *New England Journal of Medicine* (Pocock *et al.*, 1987), most trials did not even mention intended sample size, nor any policy on stopping or publication, and only five trials mentioned explicit stopping rules. More recently, we reviewed the *British Journal of Cancer*, and in 1991 and 1992 there were 16 first reports of randomised phase III trials. Eight made no mention of intended sample size or power calculations. Six of these came out with little conclusive evidence of differences between treatments, although interpretations of 'equivalence' or preference for one treatment varied. Three reports had a *post hoc* discussion of power, two of which had reached target, the third having been stopped after early results. Of the five trials which reported proper planning, one met its target, three exceeded target, and one was stopped early because of long term survival results from an associated pilot study. Only one paper mentioned any monitoring arrangements, although typically the trials were accruing patients for between three and five years, which means that data were accruing whilst new patients were still being randomised. There is no suggestion that any of these trials should have stopped any earlier, but for the trials that did not report intended sample size, it would aid interpretation to know why they stopped recruiting when they did.

### *Outline of main issues in monitoring trials*

The key decision for any potential trial is whether to start it. This means assessing the potential benefits and drawbacks of a new treatment policy compared to the standard, deciding what the benefits would need to be in order to change clinical policy, estimating the trial size needed to demonstrate such benefits, and then seeing whether such a trial is feasible. This will involve review of the existing evidence, practical considerations such as the rate at which patients with the relevant condition appear, and strategic decisions about

Received and accepted 14 June 1993.

which are the most important trials to be doing at a given time.

Having made the decision to embark on a trial, a decision to stop it prematurely should not be taken lightly. There are several good reasons (Pocock, 1983) for monitoring the progress of a trial. These include checks on protocol compliance and data management, so that remedial action can be taken while the trial is still in progress, as well as monitoring outcomes such as mortality and treatment toxicity in case it becomes necessary to stop the trial.

The main reasons for stopping a trial early are that there is a conclusive result on the main endpoint from this trial or independent trial(s), an inconclusive result on the main endpoint with no chance of achieving conclusive results, or the emergence of serious side effects on one of the treatment arms. In anticipation of such possibilities, the investigators can set up formal rules as a guide for when to stop the trial. The chief advantages of considering early stopping are that this can lead to a reduction in expected sample size, if there is a large difference in effectiveness, and the results can be disseminated sooner with consequent benefits for current patients (Fleming & Watelet, 1989). The disadvantage of early stopping is that one is left with an incomplete picture of the relative benefits of the treatments, because of less precise and possibly exaggerated estimates of effect, and because of short follow-up. Machin (1992) argues that it is unethical to stop a trial if by so doing it leaves a high level of uncertainty about the magnitude of the benefit of a new treatment.

A trial of high-dose radiotherapy with or without induction chemotherapy for stage III non-small cell lung cancer (Dillman *et al.*, 1991) stopped early because of improved survival for the group who also received chemotherapy. This decision has been criticised (Souhami *et al.*, 1991) because of the imprecision of a potentially overestimated treatment effect. The investigators (Propert *et al.*, 1991) felt it was unethical to continue to randomise patients to what they felt was an inferior treatment. The wider clinical community (Tannock & Boyer, 1992) are waiting for replication from other trials before being completely convinced. The debate over this trial highlights again the fundamental dilemma (Pocock, 1992), which is to balance the ethical considerations for the individual patients in a particular trial, which imply stopping randomisation as soon as one treatment emerges as being preferable, versus the collective ethics of the community who need accurate information of the benefits and costs of the treatment.

### *Statistical issues in monitoring trials*

For statisticians, development of plans for sequential trials has been a rich area. Early work was on fully sequential plans, which assume that the results can be updated with each new patient or pair of patients. A more recent development are group sequential plans (Pocock, 1983), which fit more neatly with the constraints of practical clinical research. These plan for a small number of analyses to occur, for example, either after fixed numbers of patients have been entered, a fixed number of deaths have been notified, or appropriately spaced in calendar time. Recent methodological work, simulating a sequential analysis based on two MRC randomised trials in patients with small-cell lung cancer, appears in this issue (Donaldson *et al.*, 1993, Whitehead, 1993). There is a growing recognition that these serve as guide-lines rather than formal stopping procedures, as the decision to stop a trial will be influenced by several factors, including both safety and efficacy considerations, and evidence external to the trial, such as the reporting of related studies.

When using the group sequential approach, a key decision is how many analyses to plan for a trial. There are big gains to be had in going from one to two analyses (Geller & Pocock, 1987), but there is little point in going above five unless an extremely large difference is anticipated, which is a very rare occurrence in cancer therapy. There are different stopping guidelines available (Emerson & Fleming, 1990), although Machin (1992) argues that Peto's simple rule of using $P<0.001$ for all but the last analysis which is done at $P<0.05$ reflects the reality of trial experience. Conventionally, all of them are structured in a similar way, with individual decision for each analysis being made at relatively stringent levels of statistical significance, which preserves the overall level of significance for the whole trial. This 'spending $P$-values' can seem a little artificial, and these rules can alternatively be interpreted, perhaps more intuitively, as a formal balancing of the current trial data with previous data and/or beliefs (Freedman & Spiegelhalter, 1989), with different rules corresponding to different prior beliefs about the relative efficacy of the treatments. Taking this approach, the investigators can formally quantify their initial position, for example, as 'sceptical' (Spiegelhalter *et al.*, 1992), which means that strong evidence from the trial is required before superiority of one of the treatments is considered to be established.

Although significance testing may be a useful guide to when to stop a trial, the primary purpose of the trial is to get an unbiased estimate of treatment effect. A problem with stopping a trial early is that a trial is likely to be stopped at a point where the estimated effects happens to be large. As a result, the estimated effect from a trial that has just stopped early will be biased. It is possible to make an adjustment for this, if the investigators can quantify their prior beliefs about the effectiveness of the treatment (Pocock & Hughes, 1989).

Particular dilemmas occur when a trial has a complex design, or similar trials are running in parallel. Such a trial, for adjuvant therapy of resected carcinoma of the colon with two treatments was reported by Moertel *et al.* (1990). Patients with stage $B_2$ cancer were randomised to observation or levamisole combined with fluorouracil. Patients with stage C were randomised to either observation, levamisole or levamisole combined with fluorouracil. The trial stopped and the results were reported because the stage C patients were performing better on the combined treatment. The authors felt able to make definite recommendations for this group, but needed more follow up for the $B_2$ patients. If this was essentially one trial, it should not have stopped because of results in a subgroup. If there were two separate trials, why was the $B_2$ trial apparently terminated early on the basis of the C patients results? As this trial had finished accrual, the implications of the decision to stop were primarily for regulatory purposes and the planning of further trials (Fleming, 1992). This raises the more general question of the best time to publish results of a trial that has finished recruitment (Altman & Machin, in press).

In fact, an overview in this journal (Gray *et al.*, 1991), shows these results to be the most dramatic out of 13 similar trials, suggesting the results in this study are by chance an overestimate of a rather more modest effect, and much larger trials are needed to make a proper assessment. Such a trial, the Adjuvant X-ray and 5FU Infusion Study (AXIS), is currently recruiting patients, and it is important that 'early' results from small trials do not compromise potentially definitive trials.

### *Practicalities to consider when setting up a trial*

Trials need to be carefully monitored, so that decisions to stop early, whether based on trial data or external evidence, can be properly made and documented.

What, in practical terms can be done? First, make a realistic assessment of possible scenarios, using general experience from cancer trials. Rigorous assessment of directly relevant trials should be carried out, using techniques such as meta-analysis (Thompson & Pocock, 1991; Parmar & Altman, in press). Subjective beliefs about the likely relative efficacy of the treatments, and the clinical benefits that would be required before a new treatment would be used routinely can also be documented at this stage, although these can be surprisingly variable, as illustrated by some work on a trial of treatment for superficial bladder cancer (Freedman & Spiegelhalter, 1983).

Mechanisms for stopping the trial must be identified, with lines of communication and responsibilities well-defined. Ideally this will involve a separate DMC, although this will not be feasible in every trial. The MRC are moving towards DMC's responsible for groups of trials in certain areas (Parmar & Machin, 1993), and there is one formally constituted to oversee the trials of the MRC Leukaemia Working Party. The Cancer Research Campaign are following a similar strategy, and now, for example, have a DMC for their breast cancer trials.

In cancer trials there will typically be relevant data on mortality, toxicity, metastases, and regression and progression. Good data management is essential, using appropriate software such as COMPACT (COMPACT Steering Group, 1991). Even with good data-management there can be time lags in information flow, and this is documented for the trial of a radiosensitiser in cervix cancer already referred to here (MRC Working Party on Advanced Carcinoma of the Cervix, 1993).

The criteria for stopping a trial should be explicit. Mortality and excess toxicity are obvious endpoints to monitor, but more complex features such as quality of life are much more difficult to assess and analyse (Fletcher *et al.*, 1992; Olschewski, in press). A particular dilemma arises when considering which endpoints to monitor because only short-term results, such as tumour response, acute morbidity and early deaths, are available quickly, whereas the real value of many trials is their potential to give information on long term survival and late morbidity. By definition, decisions to stop have to be made primarily on the early information, and it is of importance to assess to what extent this can act as surrogate information for the longer term outcomes (Ellenberg, in press). Monitoring for toxicity is always worthwhile, but monitoring for efficacy is likely to be most beneficial when mature data are accruing fast relative to the entry of new patients.

If a trial does stop early, what are the priorities? The surviving trial patients should be informed of the position, which will be much easier if they gave genuinely informed consent. When news of the neutron therapy trial's closure hit the press, the hospital switchboard was jammed with calls from concerned patients. It is a tribute to the clinicians involved that none of those calls were from trial patients, who by that time had been individually counselled. For these trials, the treatment was short-term, but for patients in trials of longer term therapy, consideration of appropriate treatment changes is an issue. The next priority should be the release of full results, quickly, via peer-reviewed journals (Pocock, 1992), although this is difficult given the current constraints of most journals.

Membership of DMC's is critical. There should be experienced, knowledgeable triallists, with both clinical and statistical expertise. Pocock (1992) suggests a clinical chairman with another clinician and a statistician, and this is the strategy adopted by the MRC (Parmar & Machin, 1993). There is sometimes a problem finding clinicians who are not already entering patients into the trials. A dilemma is whether industry employees should sit on the DMC's for their own trials. Hampton and Julian (1987) feel that DMC's should be seen to be entirely independent of the companies, and yet if there is a problem with drug safety, company people have access to other data, and expertise that may be valuable. One solution is to allow the DMC to seek advice from whoever they consider appropriate. When deciding membership of DMC's, it is worth remembering that many individuals in the pharmaceutical industry have years of experience in trials, and for trials of surgery and radiotherapy, there is no conflict of interest. However DMC's are set up, their membership should be 'public knowledge', and the balance between confidentiality of trial results, commercial or other vested interests and accountability for decision making needs to be carefully thought through.

Does every trial need to be monitored by a DMC? Fleming (1992) recommends that DMC's should be established in randomised trials diagnosed to definitively establish safety and efficacy, particularly for diseases that are life-threatening or produce irreversible morbidity. There is always a balance of costs versus risks, including the risk that the existence of a DMC may encourage early stopping. The potential benefit depends on length of time to outcome versus speed of accrual of patients. What are risks if early stopping is not considered? The major fear is the possibility of undue harm (or lack of benefits) to trial patients. If the trial is not blind, as has to be the case with many cancer trials, suspicions of a difference may arise among participating clinicians, and unplanned interim analysis may lead to a dilemma. In a trial of second-line hormone therapy versus single agent chemotherapy (Dixon *et al.*, 1992), it became evident that there was no early advantage to the group randomised to chemotherapy, although it is not clear whether this was based on clinical observation or *ad hoc* analysis. The authors say 'Having sought statistical advice, the trial was abandoned once sufficient events had occurred to allow for sufficient statistical power in its analysis'. It is far better to plan in advance.

### Conclusions

In running clinical trials, it is ethical to randomise patients while there is uncertainty as to the relative benefits of treatment, and there is indeed a duty to carry on doing so until firm evidence emerges as to which treatment is better. This is often interpreted at the level of the individual clinician, but Freedman (1987) has advocated the concept of clinical equipoise, which says that even if an individual doctor has preferences for one treatment, it is ethical to randomise while genuine uncertainty remains in the clinical community about the relative merits of treatments. When setting up a trial the protocol should always discuss arrangements for monitoring the trial, or justify lack of monitoring. The mechanisms in place should be appropriate, given the trial arrangements and the likely impact of the trial. Above all, in the excitement of the possibility of improving cancer care, triallists should also anticipate other possible outcomes.

Fifteen years ago, Pocock (1978) wrote an article for this journal on size of trials and stopping rules. This editorial says nothing fundamentally different to what he said then. However, as recent trials demonstrate, it is timely to say it again. Most trials do not need to stop early, but for the protection of the trial patients, and the future patients whose treatment may be influenced by current trials, data monitoring should be an integral part of the design of cancer clinical trials.

### References

ALTMAN, D.G. & MACHIN, D. (1993). Survival analysis. *Br. J. Cancer*, (in press).

ATKINS, H., HAYWARD, J.L., KLUGMAN, D.J. & WAYTE, A. (1972). Treatment of early breast cancer a report after ten years of a clinical trial. *Br. Med. J.*, **2**, 423–429.

BRINKLEY, D. & HAYBITTLE, J.L. (1966). Treatment of stage-II carcinoma of the female breast. *Lancet*, **2**, 291–295.

COMPACT STEERING GROUP, (1991). Improving the quality of data in clinical trials in cancer. *Br. J. Cancer*, **63**, 412–415.

DILLMAN, R.O., SEAGREN, S.L., PROPERT, K.J., GUERRO, J., EATON, W.L., PERRY, M.C., CAREY, R.W., FREI, E.F. & GREEN, M.R. (1991). A randomised trial of induction chemotherapy plus high-dose radiation versus radiation alone in stage III non-small-cell lung cancer. *New England J. Med.*, **323**, 940–945.

DIXON, A.R., JACKSON, L., CHAN, S., HAYBITTLE, J.L. & BLAMEY, R.W. (1992). A randomised trial of second-line hormone *vs* single agent chemotherapy in tamoxifon resistant advanced breast cancer. *Br. J. Cancer*, **66**, 402–404.

DONALDSON, A.N., WHITEHEAD, J., STEPHENS, R. & MACHIN, D. (1993). A simulated sequential analysis based on data from two MRC trials. *Br. J. Cancer*, **68**, 1171–1178.

ELLENBERG, S. (1993). Surrogate endpoints in clinical trials. *Br. J. Cancer*, (in press).

EMERSON, S.S. & FLEMING, T.R. (1990). Interim analyses in clinical trials. *Oncology*, **4**, 126–133.

ERRINGTON, R.D., ASHBY, D., GORE, S.M., ABRAMS, K.R., MYINT, S., BONNETT, D.E., BLAKE, S.W. & SAXTON, T.E. (1991). High energy neutron treatment for pelvic cancers: study stopped because of increased mortality. *Br. Med. J.*, **302**, 1045–1051.

FLEMING, T.R. & WATELET, L.F. (1989). Approaches to monitoring clinical trials. *J. Natl Cancer Inst.*, **81**, 188–193.

FLEMING, T.R. (1992). Evaluating therapeutic interventions: some issues and experiences. *Statistical Sci.*, **7**, 428–456.

FLETCHER, A., GORE, S.M., JONES, D.R., FITZPATRICK, R., SPIEGELHALTER, D.J. & COX, D.R. (1992). Quality of life measures in health care: design, analysis and interpretation. *Br. Med. J.*, **305**, 1145–1148.

FREEDMAN, B. (1987). Equipoise and the ethics of clinical research. *New England J. Med.*, **317**, 141–145.

FREEDMAN, L.S. & SPIEGELHALTER, D.J. (1983). The assessment of subjective opinion and its use in relation to stopping rules in clinical trials. *The Statistician*, **32**, 153–160.

FREEDMAN, L.S. & SPIEGELHALTER, D.J. (1989). Comparison of Bayesian with group sequential methods for monitoring clinical trials. *Controlled Clinical Trials*, **10**, 357–367.

GELLER, N.L. & POCOCK, S.J. (1987). Interim analyses in randomised clinical trials: ramifications and guidelines for practitioners. *Biometrics*, **43**, 213–223.

GRAY, R., JAMES, R., MOSSMAN, J. & STENNING, S.P. (1991). AXIS-a suitable case for treatment. *Br. J. Cancer*, **63**, 841–845.

HAMPTON, J.R. & JULIAN, D.G. (1987). Role of the pharmaceutical company in major clinical trials. *Lancet*, **ii**, 1258–1259.

KAAE, S. & JOHANSEN, H. (1962). Five year results: Two random series of simple mastectomy with postoperative irradiation versus extended radical mastectomy. *Amer. J. Roentgenol.*, **87**, 82–88.

MACHIN, D. (1992). Interim analysis and ethical issues in the conduct of trials. In *Introducing New Treatments for Cancer: Practical, Ethical and Legal problems*. Williams, C.J. (ed.). Wiley.

MEIER, P. (1979). Terminating a trial- the ethical problem. *Clinical Pharmacol & Therapeutics*, **25**, 633–640.

MOERTEL, C.G., FLEMING, T.R., MACDONALD, J.S., HALLER, D.G., LAURIE, J.A., GOODMAN, P.J., UNGERLEIDER, J.S., EMERSON, W.A., TORMEY, D.C., GLICK, J.H., VEEDER, M.H. & MAILLIARD, J.A. (1990). Levamisole and fluorouracil for adjuvant therapy of resected colon carcinoma. *New England J. Med.*, **322**, 352–358.

MRC WORKING PARTY ON ADVANCED CARCINOMA OF THE CERVIX, (1993). A trial of Ro 03-8799 (pimonidazole) in carcinoma of the uterine cervix: an interim report from the Medical Research Council Working Party on advanced cancer of the cervix. *Radiotherapy & Oncol.*, **26**, 93–103.

OLSCHEWSKI, M. (1993). Quality of life in clinical trials. *Br. J. Cancer*, (in press).

PARMAR, M.K.B. & ALTMAN, D. (1993). Meta-analysis. *Br. J. Cancer*, (in press).

PARMAR, M.K.B. & MACHIN, D. (1993). Monitoring clinical trials: Experience of, and proposals under consideration by, the Cancer Therapy Committee of the British Medical Research Council, in *Practical Issues in Data Monitoring of Clinical Trials*. Ellenberg, S., Geller, N., Simon, R. & Yusuf, S. (eds). *Statistics in Med.*, **12**, 497–504.

POCOCK, S.J. & HUGHES, M.D. (1989). Practical problems in interim analyses with particular regard to estimation. *Controlled Clinical Trials*, **10**, 2209S–2215S.

POCOCK, S.J., HUGHES, M.D. & LEE, R. (1987). Statistical problems in the reporting of clinical trials: A survey of three medical journals. *New England J. Med.*, **317**, 426–432.

POCOCK, S.J. (1978). Size of cancer clinical trials and stopping rules. *Br. J. Cancer*, **38**, 757–766.

POCOCK, S.J. (1983). *Clinical Trials – a Practical Approach*. Wiley: Chichester.

POCOCK, S.J. (1992). When to stop a clinical trial. *Br. Med. J.*, **305**, 235–240.

PROPERT, K.J., DILLMAN, R.O., SEAGREN, S.L. & GREEN, M.R. (1991). Chemotherapy and radiation therapy as compared with radiation therapy in stage III non-small-cell cancer. *New England J. Med.*, **323**, 1136–1137.

SOUHAMI, R.L., SPIRO, S.G. & CULLEN, M. (1991). Chemotherapy and radiation therapy as compared with radiation therapy in stage III non-small-cell cancer. *New England J. Med.*, **323**, 1136.

SPIEGELHALTER, D.J., FREEDMAN, L.S. & PARMAR, M.K.B. (1993). Applying Bayesian ideas in drug development and clinical trials, In *Methodological and Ethical Issues in Clinical Trials* Ashby, D. (ed.). *Statistics in Med.*, **12.**

TANNOCK, I.F. & BOYER, M. (1992). When is a cancer treatment worthwhile? *New England J. Med.*, **323**, 989–990.

THOMPSON, S.G. & POCOCK, S.J. (1991). Can meta-analyses be trusted? *Lancet*, **338**, 1127–1130.

WHITEHEAD, J. (1993). Interim analysis and stopping rules in cancer clinical trials. *Br. J. Cancer*, **68**, 1179–1185.